# Increasing silage maize yield and nitrogen use efficiency as a result of combined rabbit manure and mineral nitrogen fertilization

**DOI:** 10.1038/s41598-024-56669-z

**Published:** 2024-03-11

**Authors:** Andrzej Wysokinski, Monika Kożuchowska

**Affiliations:** University of Siedlce, Faculty of Agricultural Sciences, Konarskiego 2 Str., 08110 Siedlce, Poland

**Keywords:** Plant sciences, Ecology, Environmental sciences

## Abstract

Combined application of organic and mineral fertilizers is crucial to obtaining high crop yields, increasing the utilization of nutrients by plants, and limiting their dispersion, thus protecting the environment, which underscores the importance of sustainable and minimally invasive agriculture. The aim of the field experiment was to determine the effect of application of rabbit manure (RM) and mineral nitrogen (N_min_) on the dry matter (DM) yield of maize and on nitrogen content, uptake, and use efficiency (NUE). RM application was tested at levels of 0, 20, 40 and 60 t·ha^−1^, and N_min_ application at 0, 50, 100 and 150 kg·ha^−1^. Significant differences were noted in yield and in the content and uptake of nitrogen depending on both experimental factors. Increasing the application of RM and N_min_ led to an increase in the yield of harvested maize and in the content and uptake of nitrogen. In terms of DM yield and nitrogen uptake (yield of crude protein), the most beneficial fertilizer variant was 60 t·ha^−1^ RM applied together with 100 kg·ha^−1^ N_min_. The highest NUE value was obtained following application of 20 t·ha^−1^ RM together with 150 kg·ha^−1^ N_min_.

## Introduction

To meet the high and continually growing demand for food, agriculture is required to continually increase its production power. High crop yields can be obtained by providing them with access to a large amount of nutrients. This has led to the appearance of areas that have been excessively fertilized with mineral fertilizers, resulting in environmental pollution from agricultural sources. Therefore, there is a need for more efficient plant utilization of fertilizer nutrients in order to protect the environment and at the same time ensure food security^1,2^. The 4R principle should be applied: (1) the right nutrient source, (2) the right application rate, (3) the right application time, and (4) the right place of application. This will ensure adequate utilization of soil-applied nutrients by l optimizing productivity.

The use of organic fertilizers for crops reduces the amount of nutrients introduced to the soil with inorganic fertilizers. Closing the nutrient cycle on a farm is important for protection of the natural environment and also can reduce fertilizer costs.

Manure contains macro and micronutrients for plants, and land application in crop production recycles those manure nutrients. The use of nutrients in animal manure can to a large extent (for example 37.3% N, 87.6% P_2_O_5_ and 65.9% K_2_O in China) replace chemical fertilizers^3^.

Some countries (France, Spain, China, and Argentina) have large rabbit (*Oryctolagus cuniculus*) breeding industries^4,5^. In some parts of Poland the development of large-scale rabbit farming is observed as well. According to the Statistical Yearbook of Agriculture^6^, between 2010 and 2020 the number of rabbits sent to industrial slaughter in Poland increased from 290,000 to 370,000. One fattening unit rabbit produces on average 10–12 kg of manure in one cycle (about 150 g per day as the sum of faeces and urine). The population of 370,000 slaughter rabbits will therefore produce approximately 4000 tons of manure, which must be managed in an environmentally-safe manner. Rabbit manure is a fertilizer rich in organic matter and nutrients for plants and has a low or medium C/N ratio^7–10^. The average efficiency of ingested nitrogen in rabbit fattening is about 40% and drops sharply with age^11^. Thus about 60% of ingested nitrogen ends up in faeces. Calvet et al.^11^ report that urine and faeces contributed to overall nitrogen excretion in approximately the same proportions.

Rabbit manure can be used directly to fertilize crops^10,12,13^, as an ingredient in plant growth substrates^14,15^, as an adsorbent^16^, for biochar production^17,18^, and even in anaerobic fermentation to produce methane^19^.

Organic fertilizers are known to improve the physical, chemical, and biological properties of soils. These fertilizers, including rabbit manure, increase the content of organic matter^15^, pH, cation exchange capacity^20^, organic carbon content in soil, total fungi, and bacteria^21^. The use of manure as fertilizer increases the yield and quality of crops, e.g. the content of protein and minerals^15,21,22^.

Rabbit manure used in combination with chemical fertilizers has a particularly beneficial effect on crop yield and quality^10,22,23^. Combined application of nitrogen from RM (organic) and from mineral/inorganic nitrogen (from chemical fertilizers) increases the efficiency of nitrogen utilization from these fertilizers. In a pot experiment, combined application of rabbit manure and mineral nitrogen compared to the only nitrogen mineral treatment was shown to reduce the percentage of nitrogen lost from sandy and clayey soils while increasing the amount of mineral nitrogen and nitrogen present in the form of microbial biomass^24^. According to Wu and coauthors^24^, the data cannot be extrapolated to field conditions, and future research should verify the results in field conditions.

In Poland, in the years preceding the experiment, the production of silage maize systematically increased: 2010 year—395,000 ha; 2013 year—462,000 ha; 2015 year—555,000 ha; 2017 year—596,000 ha^6^. Including manures in the fertilization of silage maize can have a major impact on the success of its cultivation, affecting not only the yield, but also reducing the costs of cultivation.

The aim of the field experiment was to determine the effect of rabbit manure and supplementary application of nitrogen on the yield of silage maize, the content and uptake of nitrogen, and nitrogen use efficiency by this plant.

## Methods

### Experimental design

The field experiment was carried out in the years 2018–2019 at the Experimental Agricultural Station of the University of Siedlce (GPS, N: 52° 03′ 42.01″, E:22° 33′ 09.01″, 168 m above sea level, eastern Poland). Before setting up the experiment, 10 soil cores were taken from a 24 × 60 m area, from a 0.30 m layer, from which one soil sample was made. The soil consisted of the following fractions: 2.0–0.05 mm = 72.45%; 0.05–0.02 mm = 15.86%; 0.02–0.002 mm = 10.11%; < 0.002 mm = 1.56%. The soil pH measured in KCl 1 mol·dm^−1^ was 5.5. The content of total nitrogen (N_tot_) and organic carbon (C_org_) amounted 0.79 and 8.40 g·kg^−1^, respectively. The content of plant available phosphorus (P_av_), potassium (K_av_) and magnesium (Mg_av_) were 66, 87 and 53 mg·kg^−1^ of soil, respectively. Rainfall and air temperature recordings were obtained from the University’s own weather station (Table 1).

**Table 1:** Rainfall and air temperatures in 2018 and 2019 (weather station of the University in Siedlce).

Month	Average monthly temperatures in experimental years, °C		Total monthly rainfall in experimental years, mm	
	2018	2019	2018	2019
April	13.1	9.8	34.5	5.9
May	17.0	13.3	27.3	59.8
June	18.3	17.9	31.5	35.9
July, VII	20.4	18.5	22.4	29.7
August	20.6	19.9	24.5	43.9
September	15.9	14.2	27.4	17.4
Average/sum April–September	17.6	15.6	167.6	192.6
Average/sum May–August	19.1	17.4	105.7	169.3

The experiment was two-factorial, set up in a randomized block design in triplicate. The first factor was varied application rates of rabbit manure (RM): 0, 20, 40 and 60 t·ha^−1^. The second factor was varied application rates of mineral nitrogen (N_min_): 0, 50, 100 and 150 kg·ha^−1^. The area of each plot was 9 m^2^ (3 × 3 m). Supplementary fertilization with phosphorus and potassium was used in the treatments to obtain a nitrogen:phosphorus:potassium ratio of no less than 1:0.35:1. In the treatment without nitrogen application, phosphorus and potassium were applied in amounts corresponding to the average application rate in all fertilizer treatments. Mineral nitrogen was applied in the form of ammonium nitrate (34.0% N), phosphorus in the form of triple superphosphate (19.4% P), and potassium in the form of potassium chloride (49.8% K). All fertilizers were incorporated to the soil once in the first year (2018), prior to the experiment, 10 days before sowing the test plant. The effect on the succeeding crop was examined the following year. No fertilizer was applied for crop in 2019, and the experiment took place on the exact same plot and plot layout in both years. In both growing seasons the test plant was the ‘Tapas’ cultivar of maize (*Zea mays* L.), harvested for silage. Seeds were sown in the last 10 days of April at the rate of 100,000 per hectare, and the above-ground parts were harvested at an average height of 20 cm in the first 10 days of September. Winter triticale (*Triticosecale Wittm.* ex A. Camus) was grown in the field as a forecrop for maize. Its grain and straw were harvested in the summer of 2017. Rabbit manure contained 28.4% dry matter, total carbon 370 g·kg^−1^ DM, total nitrogen 20.6 g·kg^−1^ DM, carbon/nitrogen ratio 18.0, N-NH_4_^+^ 4.22 g·kg^−1^ DM, N-NO_3_^−^ 0.07 g·kg^−1^ DM, total phosphorus 7.06 gP·kg^−1^ DM, and total potassium 13.4 gK·kg^−1^ DM. The total amounts of nitrogen introduced to the soil and the plant available nitrogen in the treatments are shown in Table 2. According to legal regulations in Poland^25^, the total amount of nitrogen introduced to the soil with manure is converted to nitrogen available for plants (nitrogen which can actually be taken up by the plant). The amount of this form of nitrogen is calculated by multiplying the total amount of nitrogen applied by appropriate factors (multipliers), established for the next 2 years after manure application. For rabbit manure applied in spring they are 0.35 in the first year and 0.15 in the second year. Nitrogen from mineral fertilizers is assumed to be available for plants only in the first year after their application.

**Table 2:** Amounts of total nitrogen (N_tot_) and nitrogen theoretically available for plants (N_av_) in experimental treatments, kg·ha^−1^.

Treatments	Nitrogen forms	N_min_ kg·ha^−1^			
		0	50	100	150
RM (t·ha^−1^)					
0	N_tot_	0	50	100	150
	N_av_ in 2018	0	50	100	150
	N_av_ in 2019	0	0	0	0
20	N_tot_	117	167	217	267
	N_av_ in 2018	41	91	141	191
	N_av_ in 2019	17	17	17	17
40	N_tot_	233	283	333	383
	N_av_ in 2018	82	132	182	232
	N_av_ in 2019	35	35	35	35
60	N_tot_	350	400	450	500
	N_av_ in 2018	123	173	223	273
	N_av_ in 2019	53	53	53	53

### Crop samplings, measurements and calculations

Maize plants were harvested from entire plots and then weighed. Four representative plants were collected from each plot for laboratory analyses. They were first chopped into pieces 1–2 cm in length and dried to obtain a constant mass at 105 °C for determination of dry matter (DM), and then they were ground in laboratory mills. Nitrogen content was measured by a standard Kjeldahl method following wet mineralization in concentrated H_2_SO_4_^26^.

Nitrogen uptake by maize (N_up_):

$${\text{N}}_{{{\text{up}}}} = {\text{ Y }}\cdot{\text{ N}}_{{\text{c}}} ,$$

where Y is the dry mass of maize, N_c_ is the total nitrogen content (concentration) in maize’s dry mass.

Nitrogen use efficiency from total nitrogen doses (NUE_tot_):

$${\text{NUE}}_{{{\text{tot}}}} = \, \left( {{\text{N}}_{{{\text{up}}\_{\text{N}}}} - {\text{ N}}_{{{\text{up}}\_{\text{N}}0}} } \right)/{\text{N}}_{{{\text{applied}}}} \times {1}00\% ,$$

where N_up_N_ is the nitrogen uptake by maize fertilized with nitrogen, N_up_N0_ is the nitrogen uptake by maize nonfertilized with nitrogen, N_applied_ is the total amount of nitrogen applied into soil with RM and N_min_.

Nitrogen use efficiency from total nitrogen doses as the sum from 2 years (NUE_tot_2_):

$${\text{NUE}}_{{{\text{tot}}\_{2}}} = \, \left( {{\text{N}}_{{{\text{up}}\_{\text{N}}\_{2}}} - {\text{ N}}_{{{\text{up}}\_{\text{N}}0\_{2}}} } \right)/{\text{N}}_{{{\text{applied}}}} \times { 1}00\% ,$$

where N_up_N_2_ is the sum for 2 years nitrogen uptake by maize fertilized with nitrogen, N_up_N0_2_ is the sum for 2 years nitrogen uptake by maize nonfertilized with nitrogen, N_applied_ is the total amount of nitrogen applied into soil with RM and N_min_.

Nitrogen use efficiency from available nitrogen doses (NUE_av_):

$${\text{NUE}}_{{{\text{av}}}} = \, \left( {{\text{N}}_{{{\text{up}}\_{\text{N}}}} - {\text{ N}}_{{{\text{up}}\_{\text{N}}0}} } \right)/{\text{N}}_{{{\text{av}}}} \times {1}00\% ,$$

where N_up_N_ is the nitrogen uptake by maize fertilized with different doses of nitrogen, (in 1st, in 2nd, and as sum for 2 years, respectively), N_up_N0_ is the nitrogen uptake by maize nonfertilized with nitrogen. (in 1st, in 2nd, and as sum for 2 years, respectively), N_av_ is the amount of theoretically available pool of nitrogen, (from RM and N_min_ in 1st year, only from RM in 2nd year, and as the sum of nitrogen available in 1st and 2nd years, respectively).

Yield increase after application of RM (Y_i_RM_) and N_min_ (Y_i_Nmin_) separately:

$${\text{Y}}_{{{\text{i}}\_{\text{RM}}}} = {\text{ Y}}_{{{\text{RM}}}} - {\text{ Y}}_{0} ,$$

$${\text{Y}}_{{{\text{i}}\_{\text{Nmin}}}} = {\text{ Y}}_{{{\text{Nmin}}}} - {\text{ Y}}_{0} ,$$

where Y_RM_ is the yield of maize after only RM application, Y_Nmin_ is the yield of maize after only N_min_ application, Y_0_ is the yield of maize without fertilization (control object).

Percent of yield increase after application of RM (Y_i_RM_) and N_min_ (Y_i_Nmin_) separately:

$$\% {\text{Y}}_{{{\text{i}}\_{\text{RM}}}} = \, \left( {{\text{Y}}_{{{\text{i}}\_{\text{RM}}}} /{\text{Y}}_{0} } \right) \times {1}00\% ,$$

$$\% {\text{Y}}_{{{\text{i}}\_{\text{Nmin}}}} = \, \left( {{\text{Y}}_{{{\text{i}}\_{\text{Nmin}}}} /{\text{Y}}_{0} } \right) \times {1}00\% ,$$

where Y_i_RM_ is the yield increase of maize after only RM application, Y_i_Nmin_ is the yield increase of maize after only N_min_ application, Y_0_ is the yield of maize without fertilization (control object).

Yield increase after combined application of RM and N_min_ (interaction effect, Y_i_RM_Nmin_):

$${\text{Y}}_{{{\text{i}}\_{\text{RM}}\_{\text{Nmin}}}} = {\text{ Y}}_{{{\text{RM}}\_{\text{Nmin}}}} - {\text{ Y}}_{0} - {\text{ Y}}_{{{\text{i}}\_{\text{RM}}}} - {\text{ Y}}_{{{\text{i}}\_{\text{Nmin}}}} ,$$

where Y_RM_Nmin_ is the yield of maize after combined application of RM and N_min_, Y_0_ is the yield of maize without fertilization (control object), Y_i_RM_ is the increase of maize yield after only RM application, Y_i_Nmin_ is the increase of maize yield after only N_min_ application.

Percent of yield increase after combined application of RM and N_min_ (interaction effect, %Y_i_RM_Nmin_):

$$\% {\text{Y}}_{{{\text{i}}\_{\text{RM}}\_{\text{Nmin}}}} = {\text{ Y}}_{{{\text{i}}\_{\text{RM}}\_{\text{Nmin}}}} /\left( {{\text{Y}}_{{{\text{i}}\_{\text{RM}}}} + {\text{ Y}}_{{{\text{i}}\_{\text{Nmin}}}} } \right) \times {1}00\% ,$$

where Y_i_RM_Nmin_ is the yield increase after combined application of RM and N_min_, Y_i_RM_ is the increase of maize yield after only RM application, Y_i_Nmin_ is the increase of maize yield after only N_min_ application.

### Statistical analysis

The results were subjected to analysis of variance (ANOVA) with the Fisher–Snedecor distribution at a significance level of α = 0.05. Least significant difference (LSD) values at a significance level of α = 0.05 were calculated by the Tukey test. The Statistica 13.1.336.0 PL statistics package (StatSoft Inc., Tulsa, OK, USA) was used for the calculations.

Three-factor analysis of variance for data of yield, nitrogen content, uptake and NUE_tot_ were performed according to the following model:

$${\text{y}}_{{{\text{ijlp}}}} = {\text{ m }} + {\text{ a}}_{{\text{i}}} + {\text{ b}}_{{\text{j}}} + {\text{ c}}_{{\text{l}}} + {\text{ ab}}_{{{\text{ij}}}} + {\text{ ac}}_{{{\text{il}}}} + {\text{ bc}}_{{{\text{jl}}}} + {\text{ abc}}_{{{\text{ij}}}} {\text{l }} + {\text{ e}}_{{{\text{ijl}}}} ,$$

where y_ijlp_ is the value of the examined characteristic, m is the population average, a_i_ is the effect of differentiated fertilization with RM, b_j_ is the effect of differentiated fertilization with N_min_, c_l_ is the effect of year (Y), ab_ij_ is the effect of the interaction of RM x N_min_, ac_il_ is the effect of the interaction of RM × Y, bc_jl_ is the effect of the interaction of N_min_ × Y, abc_ijl_ is the effect of the interaction of RM × N_min_ × Y, e_ijl_ is the random error (numbers).

Two-factor analysis of variance for data of total NUE_tot_ as sum for 2 years and NUE_av_ calculated for nitrogen theoretically available in subsequent years of study were performed according to the following model:

$${\text{y}}_{{{\text{ijp}}}} = {\text{ m }} + {\text{ a}}_{{\text{i}}} + {\text{ b}}_{{\text{j}}} + {\text{ ab}}_{{{\text{ij}}}} + {\text{ e}}_{{{\text{ij}}}} ,$$

where y_ijp_ is the value of the examined characteristic, m is the population average, a_i_ is the effect of differentiated fertilization with RM, b_j_ is the effect of differentiated fertilization with N_min_, ab_ij_ is the effect of the interaction of RM × N_min_, e_ij_ is the random error (numbers).

Relationships between selected traits were evaluated by simple correlation analysis (α = 0.05) by the Statistica software.

### Ethical approval

The authors hereby declare that all methods were carried out in accordance with relevant guidelines.

## Results

The dry matter yield of silage maize differed between RM application rates, mineral nitrogen application rates, and years of the study (Table 3). On average for the 2 years of the study, application of increasing amounts of RM caused an increase in the dry weight of harvested maize. In the first year, the difference in yield following application of 40 and 60 t·ha^−1^ RM was not significant. In the second year, a significant increase in the amount of silage maize was obtained following application of 40 and 60 t·ha^−1^ RM, while the dose of 20 t·ha^−1^ RM did not significantly affect the yield. As in the first year, the differences between these rates were also not significant.

**Table 3:** Maize yield [DM t·ha^−1^] as a result of different RM and N_min_ application rates and years, mean values ± standard deviation (SD).

Treatments	Years		LSD_0.05_, *p*RM × Years, N_min_ × Years	Averages	LSD_0.05_, *p* RM, N_min_
	2018	2019			
RM t·ha^−1^					
0	11.3 ± 1.6 A	12.4 ± 1.4 A	1.7 *p* < 0.0001	11.8 ± 1.6 a	1.2 *p* < 0.0001
20	14.0 ± 1.7 B	13.9 ± 0.6 AB		14.0 ± 1.3 b	
40	16.9 ± 3.6 C	14.1 ± 1.1 BC		15.5 ± 3.0 c	
60	18.3 ± 3.1 C	15.7 ± 1.7 C		17.0 ± 2.8 d	
N_min_ kg·ha^−1^					
0	12.5 ± 2.5 A	13.6 ± 1.7 A	1.7 *p* = 0.0002	13.1 ± 2.2 a	1.2 *p* < 0.0001
50	14.3 ± 2.8 B	13.9 ± 1.8 A		14.1 ± 2.3 ab	
100	16.1 ± 4.1 C	14.1 ± 1.8 A		15.1 ± 3.2 bc	
150	17.5 ± 3.8 C	14.5 ± 1.7 A		16.0 ± 3.3 c	
Averages	15.1 ± 3.8 b	14.0 ± 1.7 a			
LSD_0.05_, *p* for years	0.7,* p* = 0.0016				

a,b,c,d—means for investigated factors with different lowercase letters are significantly different; A,B,C—means for interactions with different capital letters in the columns are significantly different.

On average for the 2 years of the study, increasing application of mineral nitrogen (N_min_) by 50 kg·ha^−1^ did not result in an increase in the dry weight of harvested maize between adjacent application rates (Table 3). In comparison with the control without N_min_ application, the yield was higher following application of 100 and 150 kg N_min_·ha^−1^, and in comparison with application of 50 kg N_min_·ha^−1^, the yield was higher following application of 150 kg N_min_·ha^−1^. Analysis of interactions indicates that in the first year the yield increased significantly at application of up to 100 kg N_min_·ha^−1^, while application of 150 kg N_min_·ha^−1^ did not result in a significant increase in yield. In the second year, maize yield was not significantly dependent on the amount of N_min_ applied in the first year. On average for the 2 years of the study, the yield in the second year was lower than in the first year.

The actual effect of the interaction of RM and N_min_ in determining maize yield, in comparison with the sum of the effects of RM and N_min_ applied separately, is shown in Table 4. Based on the data the effect of the interaction of RM and N_min_ can be ranked in the following ranges of percentages: 1 (> 30.0%) RM 40 t + N_min_ 150 kg; 2 (20.1–30.0%) RM 60 t + N_min_ 100 kg; 3 (10.1–20.0%) none; 4 (0.1–10.0%) RM 20 t, 40 t and 60 t, all + N_min_ 50 kg, RM 40 t + N_min_ 100 kg, RM 20 t and 60 t, both + N_min_ 150 kg; 5 (≤ 0.0%) RM 20 t + N_min_ 100 kg. The positive effects of the interaction may be due on the one hand to the increase in the mineralization rate of organic fertilizers induced by chemical nitrogen fertilizers, and on the other hand to the reduction in the dissipation of mineral nitrogen in the environment caused by organic fertilizer.

**Table 4:** Effect of the interaction of RM and N_min_ in determining maize yield (DM t·ha^−1^) and % increase in yield.

Treatments	N_min_ kg·ha^−1^								Averages	
	0		50		100		150			
	t·ha^−1^	%	t·ha^−1^	%	t·ha^−1^	%	t·ha^−1^	%	t·ha^−1^	%
RM t·ha^−1^										
0	–	–	0.9^2^	8.5^4^	1.7^2^	16.0^4^	2.4^2^	22.6^4^	1.7^2^	15.7^4^
20	2.1^1^	19.8^3^	0.1^5^	3.3^6^	− 0.3^5^	− 7.9^6^	0.3^5^	7.9^6^	< 0.1^5^	1.1^6^
40	3.1^1^	29.2^3^	0.4^5^	10.0^6^	0.3^5^	6.3^6^	1.7^5^	30.9^6^	0.8^5^	15.7^6^
60	4.7^1^	44.3^3^	0.1^5^	1.8^6^	1.4^5^	21.9^6^	0.2^5^	2.8^6^	0.6^5^	8.8^6^
Averages	3.3^1^	31.1^3^	0.2^5^	5.0^6^	0.5^5^	6.8^6^	0.7^5^	13.9^6^	0.5^5^	8.5^6^

^1^Y_i_RM_, effect of using only RM.

^2^Y_i_Nmin_, effect of using only N_min_.

^3^%Y_i_RM_, effect of using only RM.

^4^%Y_i_Nmin_, effect of using only N_min_.

^5^Y_i_RM_Nmin_, effect of using combined application of RM and N_min_ (interaction effect).

^6^%Y_i_RM_Nmin_, % effect of using combined application of RM and N_min_ (interaction effect).

The content of nitrogen in the entire dry weight of maize was significantly influenced by the RM application rates, mineral nitrogen application rates, and years of the study (Table 5). On average for the 2 years and in the second year of the study, the nitrogen content in maize increased following the application of 20 and 40 t·ha^−1^ RM, while the application of 60 t·ha^−1^ RM did not cause a significant increase in this parameter. Analysis of interactions shows that in the first year following the application of RM significantly more nitrogen was obtained in maize following the application of 40 and 60 t·ha^−1^ RM than when RM was not applied.

**Table 5:** Nitrogen content [g N·kg^−1^ DM] in maize as a result of different RM and N_min_ application rates and years, mean values ± SD.

Treatments	Years		LSD_0.05_, *p* RM × Years, N_min_ × Years	Averages	LSD_0.05_ RM, N_min_
	2018	2019			
RM t·ha^−1^					
0	10.87 ± 1.22 A	6.72 ± 0.48 A	1.15 *p* = 0.0015	8.79 ± 2.31 a	0.81 *p* < 0.0001
20	11.77 ± 1.59 AB	8.61 ± 1.70 B		10.19 ± 2.28 b	
40	12.70 ± 0.69 B	9.99 ± 0.91 C		11.35 ± 1.60 c	
60	12.63 ± 0.69 B	11.00 ± 1.71 C		11.81 ± 1.52 c	
N_min_ kg·ha^−1^					
0	11.04 ± 1.61	8.35 ± 1.59	*p* = 0.3941	9.69 ± 2.08 a	0.81 *p* < 0.0001
50	11.81 ± 1.21	8.90 ± 1.97		10.35 ± 2.18 ab	
100	12.56 ± 0.92	9.03 ± 2.19		10.80 ± 2.44 bc	
150	12.57 ± 0.88	10.04 ± 2.31		11.30 ± 2.15 c	
Averages	11.99 ± 1.32 b	9.08 ± 2.06 a			
LSD_0.05_, *p* for years	0.43, *p* < 0.0001				

a,b,c—means for investigated factors with different lowercase letters are significantly different; A,B,C—means for interactions with different capital letters in the columns are significantly different.

On average for the 2 years of the study, increasing application of mineral nitrogen (N_min_) by 50 kg·ha^−1^ did not significantly increase the content of nitrogen in the maize between adjacent rates of application. In comparison to the control without N_min_ application, maize contained more nitrogen following application of 100 and 150 kg N_min_·ha^−1^. In comparison to the application of 50 kg N_min_·ha^−1^, more nitrogen was noted in maize following application of 150 kg N_min_·ha^−1^. On average for the years of the study, the nitrogen content in maize harvested in the first year was higher than in the second year.

Nitrogen uptake by maize was significantly dependent on RM application rates, mineral nitrogen application rates, and the years of the study (Table 6). On average for the 2 years of the study, nitrogen uptake by maize increased with the RM application rate. Following application of 20, 40 and 60 t·ha^−1^ RM, nitrogen uptake was 38.2%, 72.2%, and 94.3% higher, respectively, than without RM application.

**Table 6:** Nitrogen uptake [kg N·ha^−1^] by maize as a result of different RM application rates and N_min_ and years, mean values ± SD.

Treatments	Years		LSD_0.05_, *p* RM × Years, N_min_ × Years	Averages	LSD_0.05_, *p* RM, N_min_
	2018	2019			
RM t·ha^−1^					
0	123.9 ± 29.9	83.3 ± 11.2	*p* = 0.0506	103.6 ± 30.3 a	17.0 *p* < 0.0001
20	166.8 ± 39.5	119.6 ± 24.3		143.2 ± 40.1 b	
40	215.9 ± 50.0	140.9 ± 14.9		178.4 ± 52.6 c	
60	230.5 ± 41.0	172.1 ± 27.5		201.3 ± 45.3 d	
N_min_ kg·ha^−1^					
0	141.1 ± 44.6 A	114.4 ± 29.8 A	24.0 *p* = 0.0005	127.7 ± 39.5 a	17.0 *p* < 0.0001
50	170.1 ± 43.2 B	125.4 ± 36.2 AB		147.7 ± 45.2 b	
100	204.6 ± 60.1 C	129.2 ± 40.8 AB		166.9 ± 63.3 c	
150	221.4 ± 51.9 C	146.9 ± 42.2 B		184.1 ± 59.9 d	
Averages	184.3 ± 58.0 b	129.0 ± 38.2 a			
LSD_0.05_, *p* for years	9.1, *p* < 0.0001				

a,b,c,d—means for investigated factors with different lowercase letters are significantly different; A,B,C—means for interactions with different capital letters in the columns are significantly different.

On average for the 2 years of the study, each increase in the application of mineral nitrogen (N_min_) by 50 kg·ha^−1^ caused a significant increase in nitrogen uptake by maize. Following application of 50, 100 and 150 kg N_min_·ha^−1^, nitrogen uptake was 15.7%, 30.7%, and 44.2% higher, respectively, than without application of N_min_. On average for the years of the study, nitrogen uptake by maize in the first year was 42.9% greater than in the second year.

The total nitrogen uptake as a sum from 2 years of research was significantly dependent on RM application rates or mineral nitrogen application rates (Table 7). This parameter increased with the RM application rate. The total nitrogen uptake from 2 years did not increase significantly after each increase in the mineral nitrogen dose by 50 kg·ha^−1^. Significant differences in this parameter were obtained between the objects differing in the application of 100 kg N_min_·ha^−1^ into the soil.

**Table 7:** Total nitrogen uptake (sum from 2018 and 2019) [kg N·ha^−1^] as a result of different RM and N_min_ application rates, mean values ± SD.

Treatments	N_min_ kg·ha^−1^				Averages	LSD_0.05_, *p* for RM doses
	0	50	100	150		
RM t·ha^−1^						
20	221.4 ± 7.6	263.1 ± 7.9	296.2 ± 26.9	365.2 ± 30.6	286.4 ± 57.8 a	39.9 *p* < 0.0001
40	292.8 ± 27.7	334.9 ± 21.2	372.6 ± 37.5	426.9 ± 62.9	356.8 ± 62.1b	
60	345.1 ± 5.7	385.7 ± 39.8	444.6 ± 87.4	434.9 ± 28.1	402.6 ± 59.8 c	
Averages	286.4 ± 55.8 a	327.9 ± 58.1 ab	371.1 ± 81.1bc	409.0 ± 50.1c	Interaction RM/N_min_ *p* = 0.7204	
LSD_0.05_, *p* for N_min_ doses	50.9 *p* < 0.0001					

a,b,c—means for investigated factors with different lowercase letters are significantly different.

The nitrogen use efficiency of maize was not significantly dependent on RM application rates or mineral nitrogen application rates (Table 8). The value of this parameter was significantly dependent on the year of the study. The nitrogen use efficiency of maize obtained in the first year following application of fertilizer was twice as high as in the second year.

**Table 8:** Nitrogen use efficiency [%] from total nitrogen application (N_tot_) as a result of different RM application rates and years, mean values ± SD.

Treatments	Years		*p* RM × Years, N_min_ × Years	Averages	*p* RM, N_min_
	2018	2019			
RM t·ha^−1^					
20	43.8 ± 7.8	22.1 ± 9.9	*p* = 0.1095	33.0 ± 14.1	*p* = 0.2706
40	43.9 ± 9.7	20.3 ± 3.8		32.1 ± 14.0	
60	35.1 ± 8.6	22.5 ± 5.8		28.8 ± 9.7	
N_min_ kg·ha^−1^					
50	36.4 ± 6.1	22.5 ± 7.2	*p* = 0.1933	29.5 ± 9.7	*p* = 0.3826
100	43.1 ± 9.2	19.3 ± 6.6		31.2 ± 14.5	
150	43.3 ± 11.4	23.1 ± 6.7		33.2 ± 13.8	
Averages	40.9 ± 9.4 b	21.6 ± 6.8 a			
LSD_0.05_, *p* for years	4.4, *p* < 0.0001				

a,b—means for investigated factors with different lowercase letters are significantly different.

Table 9 shows the effect of application of mineral nitrogen on NUE from RM as the sum from 2 years. NUE was shown to increase with each successive increase in application of mineral nitrogen, but the differences were not confirmed statistically. The total NUE from the 2 years of the study following application of 20 and 40 t·ha^−1^ RM was nearly identical, while following application of 60 t·ha^−1^ RM it was somewhat lower, but the significance of the differences was not confirmed statistically in this case as well. All NUE values were above 50%. An NUE value above 70% was obtained only after the application of 20 t·ha^−1^ RM and 150 kg·ha^−1^ N_min_. NUE values in the range of 60.1–70% were obtained following application of 20 t·ha^−1^ RM with supplementary application of N_min_ at 50 and 100 kg·ha^−1^; 40 t·ha^−1^ RM with supplementary application of N_min_ at all rates; and 60 t·ha^−1^ RM with supplementary application of N_min_ at 100 kg·ha^−1^. NUE values in the range of 50–60% were obtained following application of 60 t·ha^−1^ RM and N_min_ at 50 and 150 kg·ha^−1^ and following application of all levels of RM without N_min_.

**Table 9:** Total nitrogen use efficiency (sum from 2 years) [%] from total nitrogen application (N_tot_2_) as a result of different RM and N_min_ application rates, mean values ± SD.

Treatments	N_min_ kg·ha^−1^				Averages	*p* for RM doses
	0	50	100	150		
RM t·ha^−1^						
20	50.3 ± 6.5	60.2 ± 4.7	61.6 ± 12.4	75.9 ± 11.5	62.0 ± 12.4	*p* = 0.3396
40	55.7 ± 11.9	60.8 ± 7.5	62.9 ± 11.2	68.9 ± 16.4	62.1 ± 11.5	
60	52.1 ± 1.6	55.7 ± 9.9	62.6 ± 19.4	54.4 ± 5.6	56.2 ± 10.5	
Averages	52.7 ± 7.2	58.9 ± 7.1	62.4 ± 12.8	66.4 ± 14.1	Interaction RM/N_min_ *p* = 0.6158	
*p* for N_min_ doses	*p* = 0.0829					

No significant interactions were found between different RM and N_min_ rates in the case of yield, content and nitrogen uptake (as an average and sum for 2-years) by maize.

The mean values of the examined property for the two-way interactions RM × N_min_ presented in Table 10 were not significant—all *p*-values > 0.05.

**Table 10:** Level of RM × N_min_ interaction for yield data, nitrogen content and uptake, and NUE_tot_ value, mean values ± SD.

Treatments	N_min_ kg·ha^−1^			
	0	50	100	150
RM t·ha^−1^				
0				
Yield	10.6 ± 1.9	11.5 ± 1.2	12.3 ± 1.0	13.0 ± 1.4
N content	7.88 ± 1.59	8.77 ± 2.52	9.09 ± 2.50	9.43 ± 2.79
N uptake	81.3 ± 7.7	99.2 ± 23.8	111.0 ± 28.9	123.1 ± 40.1
20				
Yield	12.7 ± 1.0	13.7 ± 0.4	14.1 ± 0.6	15.4 ± 1.3
N content	8.84 ± 1.56	9.65 ± 1.87	10.49 ± 2.64	11.78 ± 2.31
N uptake	110.7 ± 11.8	131.5 ± 22.8	148.1 ± 38.2	182.6 ± 44.3
NUE_tot_	–	30.1 ± 12.1	30.8 ± 15.9	38.0 ± 15.3
40				
Yield	13.7 ± 0.7	15.0 ± 1.3	15.7 ± 2.3	17.8 ± 4.7
N content	10.72 ± 1.74	11.14 ± 1.64	11.67 ± 2.03	11.85 ± 0.96
N uptake	146.4 ± 24.6	167.4 ± 33.1	186.3 ± 56.2	213.5 ± 70.4
NUE_tot_	–	30.4 ± 10.4	31.5 ± 15.8	34.5 ± 17.4
60				
Yield	15.3 ± 1.6	16.3 ± 2.4	18.4 ± 4.0	17.9 ± 2.0
N content	11.33 ± 1.62	11.84 ± 1.52	11.93 ± 1.97	12.16 ± 1.19
N uptake	172.6 ± 24.8	192.6 ± 34.0	222.3 ± 68.2	217.4 ± 33.3
NUE_tot_	–	27.9 ± 7.7	31.3 ± 14.5	27.2 ± 6.0
*p* value for interaction RM × N_min_, and RM × N_min_ × Year				
Yield	RM × N_min_ = 0.7483, RM × N_min_ × Year = 0.3647			
N content	RM × N_min_ = 0.4938, RM × N_min_ × Year = 0.3799			
N uptake	RM × N_min_ = 0.5563, RM × N_min_ × Year = 0.7202			
NUE_tot_	RM × N_min_ = 0.5214, RM × N_min_ × Year = 0.7663			

NUE_av_ values calculated separately for each year for nitrogen theoretically available for plants were not significantly dependent on the level of RM application in either year or on the amount of N_min_ applied in the first year (Table 11). In the second year of the study, application of 0, 50 and 100 kg·ha^−1^ N_min_ together with RM did not significantly influence NUE_av_ from the pool of theoretically available nitrogen applied in the form of RM. The highest value for this parameter was obtained following the application of N_min_ at 150 kg·ha^−1^ together with RM. NUE_av_ values calculated for N theoretically available for plants in total for the 2 years of the study were not significantly dependent on either the RM or N_min_ application rate.

**Table 11:** Nitrogen use efficiency [%] from nitrogen theoretically available for plants (presented in Table 2) in each year of the study as a result of different RM and N_min_ application rates, mean values ± SD.

Years of study	Treatments	RM, t·ha^−1^			Averages	LSD_0.05_, *p* for N_min_ doses
		20	40	60		
2018	N_min_ kg·ha^−1^					*p* = 0.0552
	0	87.3 ± 4.6	97.6 ± 8.8	88.2 ± 3.2	91.0 ± 5.5	
	50	67.4 ± 8.9	83.9 ± 3.4	77.4 ± 5.9	76.2 ± 6.1	
	100	68.2 ± 5.4	81.0 ± 11.3	82.1 ± 12.9	77.1 ± 9.2	
	150	70.2 ± 0.7	79.9 ± 13.0	57.4 ± 5.0	69.1 ± 11.4	
	Averages	73.3 ± 9.2	85.6 ± 10.0	76.3 ± 7.7	Interaction RM/N_min_ *p* = 0.8180	
	*p* for RM doses	*p* = 0.1688				
2019	N_min_ kg·ha^−1^					99.3 *p* = 0.0055
	0	135.2 ± 21.1	143.3 ± 20.9	139.8 ± 19.6	139.4 ± 18.1a	
	50	230.4 ± 121.8	175.7 ± 43.9	168.3 ± 32.5	191.5 ± 72.9ab	
	100	220.0 ± 90.1	178.7 ± 26.6	186.7 ± 84.3	195.1 ± 65.9ab	
	150	403.1 ± 178.6	225.6 ± 40.8	218.3 ± 37.4	282.3 ± 130.2b	
	Averages	247.2 ± 142.8	180.8 ± 42.4	178.3 ± 51.9	Interaction RM/N_min_ *p* = 0.3841	
	*p* for RM doses	*p* = 0.0614				
For N available in both years	N_min_ kg·ha^−1^					*p* = 0.4688
	0	101.4 ± 13.1	111.3 ± 23.7	103.7 ± 3.2	105.4 ± 14.4	
	50	93.0 ± 7.3	103.2 ± 12.7	98.7 ± 17.6	98.3 ± 12.3	
	100	84.5 ± 17.0	96.8 ± 17.3	102.2 ± 21.7	94.5 ± 21.4	
	150	97.4 ± 14.7	99.0 ± 23.6	83.5 ± 8.6	93.3 ± 16.3	
	Averages	94.1 ± 13.2	102.6 ± 17.9	97.0 ± 18.0	Interaction RM/N_min_ *p* = 0.8513	
	*p* for RM doses	*p* = 0.4978				

a,b—means for investigated factors with different lowercase letters are significantly different.

Correlation analysis revealed significant relationships between total and available nitrogen doses, and: silage maize yield, nitrogen content, nitrogen uptake (Table 12). Nitrogen use efficiency in 2019 and NUE as sum from 2018 and 2019 were significantly correlated with total nitrogen doses.

**Table 12:** Linear correlation coefficients between N_tot_ and N_av_ rates and selected properties of silage maize, values marked with * are important (α< 0.05).

Specification	N_tot_ dose	N_av_ dose
Yield in 2018	0.875*	0.824*
Yield in 2019	0.688*	0.568*
Yield, as the sum 2018 + 2019	0.862*	0.849*
N content in 2018	0.671*	0.696*
N content in 2019	0.829*	0.715*
N uptake in 2018	0.872*	0.870*
N uptake in 2019	0.899*	0.770*
N uptake, as the sum 2018 + 2019	0.927*	0.872*
NUE_tot_ in 2018	0.027	–
NUE_tot_ in 2019	0.570*	–
NUE_tot_ as the sum 2018 + 2019	0.308*	–

## Discussion

Animal excrements (faeces and urine) are traditionally used to fertilize soil and plants. They owe their value as fertilizer to high content of organic matter and nutrients for plants. One of the most important nutrients for plants contained in these fertilizers is nitrogen^8,27^. Organic forms of nitrogen applied to the soil gradually become available for plants^28^. Mineralization of organic compounds from these fertilizers, spread out over time, causes the gradual release of nitrogen in available forms, which enables a regular supply of this macronutrient to plants. In the case of chemical fertilizers, the entire pool of nitrogen is usually available for plants immediately after their application, but decreases significantly in the later stage of their growth^29^. This is the result of nitrogen being taken up by plants and dissipated in the environment. The combined application of these fertilizers limits the dissipation of nitrogen from mineral fertilizers in the environment^30^. This may be the result of biological sorption of mineral nitrogen (its immobilization process). Fertilization based on the combined application of organic fertilizers and nitrogen mineral fertilizers can ensure an optimal supply of nitrogen to plants^31^ and may result in higher crop yields^30,32^. The present study showed that the biomass yield of maize increased in the first year after application of RM up to 40 t·ha^−1^ and N_min_ up to 100 kg·ha^−1^. Further increases to 60 t·ha^−1^ RM and 150 kg·ha^−1^ N_min_ did not lead to a significant increase in the yield of maize. In that second year of the study, an increase in yield in comparison to the treatment without RM application was obtained following application at the two highest rates (40 and 60 t·ha^−1^). In that year, varied application of N_min_ did not significantly differentiate the yields of the test plant, which is consistent with the idea of the rapid but short-term fertilizing effect of chemical nitrogen fertilizers. The dry matter yield per kg of applied nitrogen decreases as the rate of application increases. Nitrogen applied in small amounts is then the major growth-limiting factor. Increasing the supply of nitrogen reduces productivity of applied N, because the yield is determined by factors other than the limiting nutrient^33,34^.

An important element of the experiment was the determination of the most beneficial proportion of rabbit manure and mineral nitrogen application for the yield of the test plant, nitrogen content and uptake, and NUE. The study showed that the most favourable proportion in terms of the biomass yield of maize was 60 t·ha^−1^ RM and 100 kg·ha^−1^ N_min_, which introduced a total of 450 kg·ha^−1^ nitrogen into the soil, while the amount of nitrogen theoretically available for plants in the first and second year was 223 and 53 kg·ha^−1^, respectively. Other researchers reported that for cultivation of forage maize in semi-arid areas, the optimal application rate of nitrogen in chemical fertilizer, and thus nitrogen available for plants, was 210 kg·ha^−1^, but that was the highest rate tested in the study^34^. Correlation analysis (p < 0.05) shows that the silage maize yields were significantly dependent on the application rates of nitrogen: total (r =  + 0.862) and theoretically available for plants (r =  + 0.849). Increasing maize yields caused by increasing nitrogen application rates is a common phenomenon and consistent with researchers’ expectations^35,36^. Good supply of nitrogen to plants promotes faster photosynthesis by increasing capture of sunlight by crops and the efficiency of conversion to biomass, thus improving biomass production^37^. Photosynthesis activity is associated with nitrogen content in leaves^38^. The amount of dry matter of silage maize increases with the level of nitrogen in the leaves^33^. Combined application of organic and chemical nitrogen fertilizers has proven very effective in crops of other plants. In potato, the ideal combination for optimizing yield has been shown to be, on average, 31 t·ha^−1^ of organic manure and 187.5 kg·ha^−1^ of nitrogen fertilizer^39^. Similar fertilization systems based on partial replacement of mineral nutrients for plants with nutrients applied in the form of organic fertilizers, including rabbit manure, have resulted in similar or even higher maize yields than inorganic fertilizer applied at the same nitrogen:phosphorus:potassium rate^40^.

Increasing the amount of nitrogen introduced to the soil caused an increase in its content in the biomass of maize as well as its uptake by the plant. Converting nitrogen content to crude protein content (CP) (× 6.25) and nitrogen uptake to crude protein yield (CPY) shows that application of RM at 20–60 t·ha^−1^ resulted in CP from 7.4 to 7.9% in the biomass of maize grown in the first year after fertilizer application, and CPY from 1042 to 1441 kg·ha^−1^. In the second year following application of RM at 20–60 t·ha^−1^, CP ranged from 5.4 to 6.9%, and CPY from 748 to 1076 kg·ha^−1^. The values obtained in the first year after fertilizer application correspond to the typical protein content in maize silage^41,42^. Analysis of the interaction of RM and N_min_ application shows that the highest average CP content from the 2 years (7.6%) was obtained following the combined application of RM and N_min_ at the highest rates, i.e. 60 t·ha^−1^ RM and 150 kg·ha^−1^ N_min_, which introduced a total of 500 kg·ha^−1^ N to the soil, including 273 and 53 kg·ha^−1^ of nitrogen theoretically available for plants in the first and second year, respectively. However, the highest average crude protein yield from the 2 years (1389 kg·ha^−1^) was obtained following the combined application of 60 t·ha^−1^ RM and 100 kg·ha^−1^ N_min_. On this treatment into soil was applied 50 kg less total and theoretically available nitrogen than in the case of the highest nitrogen application. Ma and coauthors^34^ tested nitrogen application rates from 70 to 210 kg·ha^−1^ in chemical fertilizer and obtained 5.2% to 7.0% protein content in forage maize. In the present study, the average crude protein content in the maize biomass was slightly higher. Literature data indicate that it is possible to obtain even higher CP content in forage maize^43^. Following application of N_min_ alone at 50–150 kg·ha^−1^, CP content ranged from 5.5 to 5.9%, while following application of RM alone at 20–60 t·ha^−1^, CP content ranged from 5.5 to 7.1%. After combined application of RM and N_min_ in all combinations tested, CP ranged from 6.0 to 7.6%. Correlation analysis (p < 0.05) indicates that the nitrogen content and, consequently, the crude protein content in the first and second year after fertilizer application was significantly correlated with the total amount of nitrogen applied (r =  + 0.671 and r =  + 0.829, respectively), as well as with the amount of nitrogen theoretically available for plants (r =  + 0.696 and r =  + 0.715, respectively). Nitrogen uptake and, consequently, crude protein yield in the first and second year after fertilization was also significantly correlated with the total nitrogen applied (r =  + 0.872 and r =  + 0.899, respectively), as well as with the amount of nitrogen theoretically available for plants (r =  + 0.870 and r =  + 0.770, respectively). This means that as the level of nitrogen application increased (total and theoretically available for plants), uptake of nitrogen and its accumulation in the maize biomass increased. This translates directly to the NUE values, which were generally somewhat smaller following application of higher levels of nitrogen, but did not differ significantly depending on the levels of RM and N_min_ application. However, literature data indicate that the NUE value significantly decreases as N application increases^34,44^. NUE values ranged from 35.1 to 43.9% in the first year following application of RM and N_min_, and from 19.3 to 23.1% in the second year. These are higher values than in other studies, in which NUE from urea applied at 70–210 kg·ha^−1^ nitrogen was within the range of 24.4–31.7%^34^. Other researchers obtained a significant decrease in nitrogen recovery efficiency from various levels of nitrogen application after replacing inorganic nitrogen with organic nitrogen (applied with manure) at a level of at least 50%^45^. In that study, the highest value of this parameter was obtained following application of inorganic and organic nitrogen in a 0.75/0.25 ratio (about 40–50% in maize cultivation). Another study found that the optimal level of replacement of synthetic nitrogen with organic nitrogen, leading to an increase in NUE, was 44%^46^. The NUE values above 60% obtained in the present study following application of RM at 20 t·ha^−1^ (117 kg·ha^−1^ total nitrogen, 41 kg·ha^−1^ theoretically available nitrogen) and 40 t·ha^−1^ (233 kg·ha^−1^ total N, 82 kg·ha^−1^ theoretically available nitrogen) with supplementary application of N_min_ at 50–150 kg·ha^−1^ and following application of 60 t·ha^−1^ RM (350 kg·ha^−1^ total nitrogen, 123 kg·ha^−1^ theoretically available nitrogen) with 100 kg·ha^−1^ N_min_ indicate an appropriate level of nitrogen use by the plants and are in line with current sustainable development strategies for the agricultural sector. Fertilization regimes based on balanced synthetic nitrogen and manure are ideal for sustainable nitrogen management^47^, increasing NUE^48^. Calculation of NUE for the pool of nitrogen theoretically available for plants (NUE_av_) sheds new light on NUE values. The NUE_av_ values obtained in the first year, most often in the range of 60–80%, are indicative of incomplete use of the pool of nitrogen theoretically available for plants. This may have been due to unfavourable moisture conditions during the growing season. The NUE_av_ values for the pool of nitrogen theoretically available for plants in the second year, exceeding 100% in each case, and in some cases even 200%, are indicative of greater nitrogen availability for plants than was predicted. They may also indicate that the pool of available nitrogen unused in the first year was used in the second year. NUE_av_ values for the pool of nitrogen theoretically available for plants in total for both years of the study was most often over 90%, and in five cases exceeded 100% (on average 98%), indicating total or nearly total utilization of this pool of nitrogen. It also indicates that the factors chosen to convert the total amount of nitrogen introduced to the soil with manure to nitrogen available for plants were well-selected^25^.

## Conclusion

Given the dry matter yield of maize, nitrogen uptake (crude protein yield), nitrogen use efficiency from applied nitrogen, and the effect of the interaction of organic and mineral fertilizer, fertilization of maize for silage with rabbit manure at up to 40 t·ha^−1^ and supplementary application of mineral nitrogen in amounts up to 150 kg·ha^−1^ is justified. When application of rabbit manure is increased to 60 t·ha^−1^, application of N_min_ should be decreased so as not to exceed 100 kg·ha^−1^. These fertilizer combinations introduce up to 232 kg·ha^−1^ of nitrogen theoretically available for plants to the soil in the first growing season, and the NUE_av_ from this pool of nitrogen is about 80%. The values of all parameters tested (yield, nitrogen content and uptake, NUE_tot_ and NUE_av_) indicate the need for additional application of nitrogen for crops grown in the second year following application of RM, but after taking into account the small amount of available nitrogen remaining after application of RM in the first year.

## Data Availability

The datasets generated and analysed during the current study are not publicly available due to they are the authors’ own data, but are available from the corresponding author on reasonable request.
